# Different miRNAs Related to *FBXW7* Mutations or High Mitotic Indices Contribute to Rectal Neuroendocrine Tumors: A Pilot Study

**DOI:** 10.3390/ijms24076329

**Published:** 2023-03-28

**Authors:** Ho Suk Kang, Ha Young Park, Hyun Lim, Il Tae Son, Min-Jeong Kim, Nan Young Kim, Min Jeong Kim, Eun Sook Nam, Seong Jin Cho, Mi Jung Kwon

**Affiliations:** 1 Department of Internal Medicine, Hallym University Sacred Heart Hospital, Hallym University College of Medicine, Anyang 14068, Republic of Korea; 2 Department of Pathology, Busan Paik Hospital, Inje University College of Medicine, Busan 47392, Republic of Korea; 3 Department of Surgery, Hallym University Sacred Heart Hospital, Hallym University College of Medicine, Anyang 14068, Republic of Korea; 4 Department of Radiology, Hallym University Sacred Heart Hospital, Hallym University College of Medicine, Anyang 14068, Republic of Korea; 5 Hallym Institute of Translational Genomics and Bioinformatics, Hallym University Medical Center, Anyang 14068, Republic of Korea; 6 Department of Surgery, Kangdong Sacred Heart Hospital, Seoul 05355, Republic of Korea; 7 Department of Pathology, Kangdong Sacred Heart Hospital, Seoul 05355, Republic of Korea; 8 Department of Pathology, Hallym University Sacred Heart Hospital, Hallym University College of Medicine, Anyang 14068, Republic of Korea

**Keywords:** miRNAs, neuroendocrine tumor, rectum, FBXW7, miR-769-5p, miR-3934-5p

## Abstract

Recent studies suggest that miRNA may be involved in the development of rectal neuroendocrine tumors (NETs). We explored the frequency of clinicopathologically relevant mutations and miRNA expression in rectal NETs to examine molecular profiles related to prognosis and behavior. Twenty-four eligible specimens with endoscopically excised rectal NETs were selected. Next-generation sequencing and an miRNA expression assay were used to evaluate the expression profile relevant to common genetic mutations in rectal NETs. Kyoto Encyclopedia of Genes and Genomes analysis predicted that the possible target signaling pathways were correlated with dysregulated miRNAs. Nineteen rectal NETs harbored more than one mutation in the 24 cancer-related genes. Seven miRNAs (hsa-miR-769-5p, hsa-miR-221-3p, hsa-miR-34a-5p, hsa-miR-181c-5p, hsa-miR-1246, hsa-miR-324-5p, and hsa-miR-361-3p) were significantly down-regulated in tumors harboring the *FBWX7* mutation. Unsupervised hierarchical clustering analysis showed that up-regulation of these seven miRNAs may result in high mitotic indices, indicating the role of miRNAs in tumor progression. Among the down-regulated miRNAs, hsa-miR-769-5p was strongly correlated with extracellular matrix–receptor interaction and lysine degradation. Among the clinicopathological factors, up-regulated hsa-miR-3934-5p was linked to an increased mitotic count. No change in miRNA expression was associated with a tumor size >1 cm, lymphovascular invasion, or Ki-67 index. In summary, we identified different miRNA signatures involved in *FBXW7* mutations or high mitotic indices in rectal NETs, which may play a critical role in tumor behavior.

## 1. Introduction

Neuroendocrine tumors (NETs) are a heterogeneous group of tumors originating from neuroendocrine cells and harbor malignant potential [1,2]. The incidence of NETs is becoming more common worldwide, and their characteristics and behavior can vary widely [1,2]. The incidence of gastrointestinal NETs depends on the primary tumor site, with the greatest prevalence in the rectum [3,4,5]. Rectal NETs are the most frequent in Asia, including the Republic of Korea, taking up 60–89% of all gastroenteropancreatic NETs [3,4,5]. In particular, a nine-fold increase in incidence has been discovered in the Republic of Korea [5]. Most rectal NETs are small (<10 mm) and have a favorable 5-year overall survival rate of 88% [6]. However, its prognostic implications are exceedingly discussed because the tumor behavior varies according to the grade, size, invasion depth, and lymphovascular invasion (LVI) [7,8,9,10,11,12,13,14]. Moreover, its incidence may reflect ethnic or geographical differences, as rectal NETs develop predominantly in Asians. Midgut NETs, including the small intestine, occur more in white and less in Asian patients [7,8,9,10,11,12,13,14]. Virtually, the genetic mutations observed in NETs diverge depending on their anatomic location [15]. Pancreatic NETs have been illustrated to carry somatic mutations in *MEN1*, *DAXX*, *ATRX*, *PTEN*, and members of the mTOR signaling pathway [16], whereas gastrointestinal NETs have been reported to harbor *CDKN1B* mutations [15,17]. The inactivation of *RB1* and *TP53* has been described in so-called neuroendocrine carcinomas rather than NETs [16]. However, this genetic information is exclusively based on the European population [15,16]. Our previous study involving 69 rectal cases from single institutional samples in Korea demonstrated that one-third of well-differentiated, grade 1 or 2 rectal NETs, frequently carry *TP53* (10.1%) or *FBXW7* (7.2%) mutations [18]. This high prevalence of *TP53* mutations differs from the European population-based molecular results [18]. There has been scarce information on the molecular profiling of rectal NETs in the Korean population, wherein piecemeal molecular characterization may limit the explanation of the spectrum of behavior in rectal NETs [18]. Therefore, there is an inevitable need to identify key regulators that could influence the distinct molecular features of rectal NETs in the Korean population.

Epigenetic regulation of gene expression, particularly through miRNA, is an important research topic linked to cancer development [19]. miRNAs are small noncoding RNA molecules that regulate gene expression at the post-translational level by suppressing target mRNAs [20]. Gene silencing by miRNAs governs diverse physiological and pathological steps, which can be used in various cancer classifications as informative tissue markers [20]. However, studies on miRNA expression profiles in NETs have yielded conflicting results [21,22,23,24], making it challenging to identify key tissue markers [25]. For example, a previous study discovered that miR-885-5p could be an early prognostic indicator for LVI in rectal NETs [21]. However, this study did not investigate the connection between miR-885-5p and tumor grade. A more recent study in Japan examined seven rectal NET tissues and found overexpression of the miR-144/miR-451 cluster in NETs of grade 1 with LVI [22]. In another study, higher expression of miR-19a and miR-96 was found in metastatic tissue than in primary tumors of the small intestine and colon; however, the sample size was limited [23]. A meta-analysis of 22 studies found inconsistent miRNA signatures in gastric, pancreatic, small intestinal, and colorectal NETs [24]. miR-222 was associated with regulated p27KIP1 in gastric NETs, while miR-21 and miR-144 showed variable up- and down-regulation in pancreatic NETs depending on the analysis method [24]. No consistent miRNA signatures were identified in small intestinal or colorectal NETs [24]. Nevertheless, a lack of data on rectal NETs requires further validation of rectal NETs in the Korean population. In this study, the Nanostring nCounter Human v3 miRNA Expression assay screens 800 miRNAs, representing one of the vast miRNA assays as high-throughput techniques, out of date.

Therefore, we screened differentially expressed miRNAs clinically or pathologically relevant to the specific genetic mutations involved in rectal NETs based on the tumor grade to identify miRNA biomarkers related to prognosis and tumor behavior.

## 2. Results

### 2.1. Baseline Characteristics and Genetic Alterations of Rectal NETs

Overall, 24 patients (17 male and seven female) with a median age of 48.5 years (34–73 years) were included (Table 1). The average tumor size ranged from 7.62 ± 2.81 mm (5 to 15 mm). According to the World Health Organization (WHO) classification, 16 patients showed grade 1 NETs, and eight exhibited grade 2 NETs. Microscopic examination with D2-40 and hematoxylin and eosin (H&E) staining allowed us to observe seven LVIs (29.2%, 7/24). After next-generation sequencing (NGS) and variant analysis, 19 rectal NETs (79.2%, 19/24) revealed more than one mutation in the 24 cancer-related genes: *TP53* (7/24, 29.2%), *FBXW7* (5/24, 20.8%), *CDKN2A* (4/24, 16.7%), *PTEN* (4/24, 16.7%), *ATM* (3/24, 12.5%), *EGFR* (3/24, 12.5%), *AKT1* (2/24, 8.3%), *ALK* (2/24, 8.3%), *IDH1* (2/24, 8.3%), *KIT* (2/24, 8.3%), *KRAS* (2/24, 8.3%), *PIK3CA* (2/24, 8.3%), *RET* (2/24, 8.3%), *SMARCB1* (2/24, 8.3%), *VHL* (2/24, 8.3%), *BRAF* (1/24, 4.1%), *CTNNB1* (1/24, 4.1%), *ERBB2* (1/24, 4.1%), *EZH2* (1/24, 4.1%), *FLT3* (1/24, 4.1%), *HNF1A* (1/24, 4.1%), *SMAD4* (1/24, 4.1%), *SMO* (1/24, 4.1%), and *STK11* (1/24, 4.1%). *TP53*, *FBXW7*, *CDKN2A*, and *PTEN* mutations were the most frequently found in rectal NETs.

### 2.2. Screening of Candidate miRNAs Associated with Specific Genetic Mutations

We screened the expression of 584 miRNA relevant to the aforementioned 24 genetic mutations in mutated and wild-type tumors. The results revealed that *FBXW7* was the only candidate gene relevant to miRNAs differentially expressed between mutated and wild-type NETs. When comparing the miRNA expression levels of 19 tumors harboring *FBXW7* wild-type with 5 tumors carrying *FBXW7* mutations, we observed seven down-regulated miRNAs (hsa-miR-221-3p, hsa-miR-769-5p, hsa-miR-181c-5p, hsa-miR-34a-5p, hsa-miR-1246, hsa-miR-324-5p, and hsa-miR-361-3p) in *FBXW7*-mutant tumors compared to *FBXW7*-wild-type tumors (all *p* < 0.001, Table 2). Among them, hsa-miR-769-5p was the most correlated with *FBXW7* mutation (false discovery rate [FDR] < 0.05).

**Figure 1:**
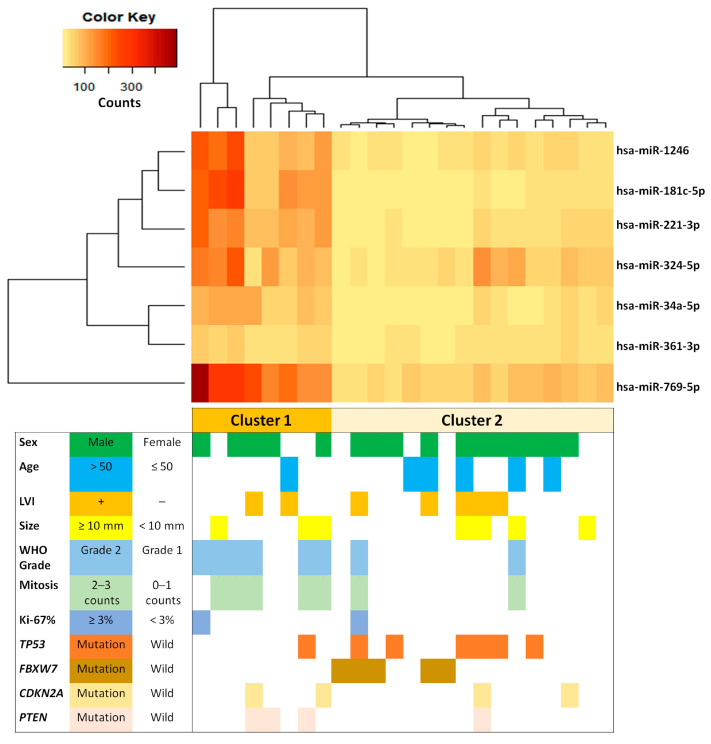
Hierarchical clustering map of differentially regulated miRNAs between *FBXW7* wild-type and mutated rectal neuroendocrine tumors. Each row represents one miRNA, and each column represents one sample. Red and light yellow indicate the high expression level above the mean and the low expression level below the mean.

Then, unsupervised hierarchical clustering analysis was performed to compare the expression profiles of the dysregulated seven miRNAs (Figure 1). The analysis separated the tumors into two main clusters according to miRNAs being up- or down-regulated. The cluster with up-regulated miRNA expression tended to correlate with NETs with higher mitotic indices, grade 2, wild-type *FBXW7*, and wild-type *TP53*. In contrast, the cluster with down-regulated miRNAs tended to correlate with NETs with lower mitotic indices, grade 1, *FBXW7* mutation, and *TP53* mutation. These findings indicated distinct miRNA differential expression (hsa-miR-769-5p, hsa-miR-221-3p, hsa-miR-34a-5p, hsa-miR-181c-5p, hsa-miR-1246, hsa-miR-324-5p, and hsa-miR-361-3p) between the tumor groups with high mitotic indices, grade 2, wild-type *FBXW7*, and wild-type *TP53* and other tumor groups with low mitotic indices, grade 1, *FBXW7* mutation, and *TP53* mutation.

### 2.3. miRNAs Related to Clinical and Pathological Characteristics

Evaluation of 584 miRNA expression profiles between clinical and pathological factors, including sex, age (≤50 vs. >50), tumor size (<10 mm vs. ≥10 mm), LVI, Ki-67 index (<3% vs. ≥3%), and WHO grade (grade 1 vs. grade 2), showed no significant differences among miRNA expression levels except for the mitotic index.

When comparing the miRNA expression profiles between tumors with either low (0–1) or high (≥2) mitotic indices, we identified five differentially expressed miRNAs between the two groups: miR-3934-5p was significantly up-regulated, and miR-153-3p, miR-4286, miR-223-3p, and miR-129-5p were down-regulated in the high mitotic index group. After adjustment, only miR-3934-5p was statistically significant (Table 3). However, miR-3934-5p, related to a high mitotic index, was not correlated with the miRNA expression profiles related to *FBXW7* mutations (*p* > 0.99999).

### 2.4. Identification of Potential Functional Pathways Related to miRNAs

**Figure 2:**
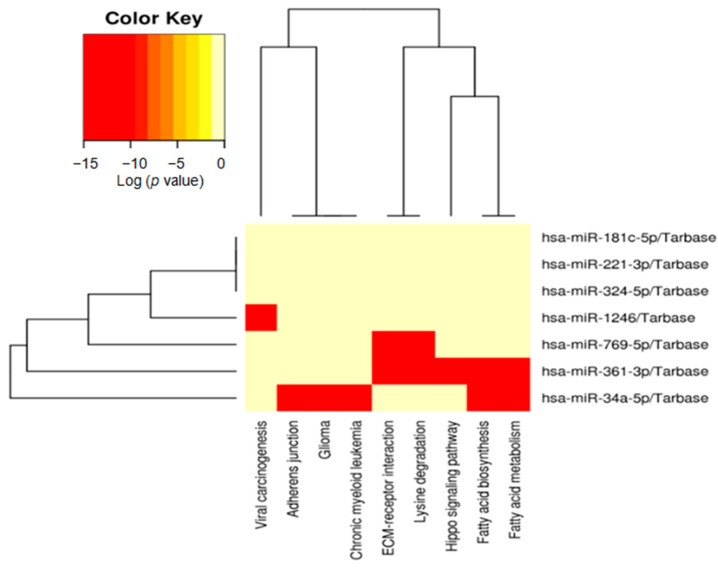
KEGG pathway heatmaps reveal that miRNAs are significantly differentially expressed in *FBXW7*-mutated rectal neuroendocrine tumors.

We tested Kyoto Encyclopedia of Genes and Genomes (KEGG) pathway maps with the mentioned miRNAs. Seven significantly differentially expressed miRNAs correlated with *FBXW7* mutational status (hsa-miR-769-5p, hsa-miR-34a-5p, hsa-miR-181c-5p, hsa-miR-324-5p, hsa-miR-221-3p, hsa-miR-361-3p, and hsa-miR-1246). This analysis enriched nine possible pathways involved in fatty acid biosynthesis, extracellular matrix (ECM)-receptor interaction, fatty acid metabolism, lysine degradation, viral carcinogenesis, the adherens junction, chronic myeloid leukemia, and glioma and the Hippo signaling pathway to identify potential functionally connected pathways between miRNAs and target genes (Table 4). Specifically, ECM-receptor interactions and lysine degradation were significantly correlated with hsa-miR-769-5p (Figure 2).

**Figure 3:**
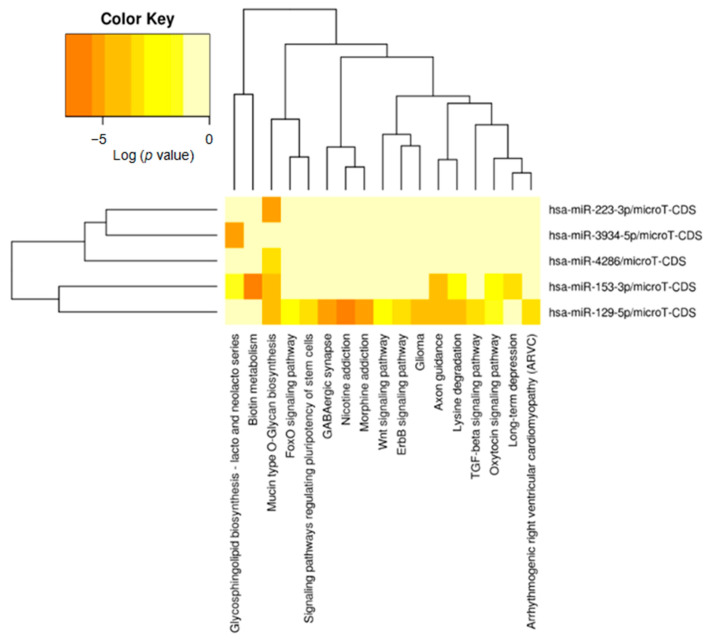
KEGG pathway heatmaps of differentially expressed miRNAs with significantly different expression in mitotic counts (*p* < 0.01).

Furthermore, we produced additional KEGG pathway maps with five differentially expressed miRNAs that correlated with a high mitotic index (miR-3934-5p, miR-153-3p, miR-4286, miR-223-3p, and miR-129-5p). This analysis enriched 17 possible pathways in which the glycosphingolipid biosynthesis-lacto and neolacto series were significantly correlated with hsa-miR-3934-5p (Figure 3).

## 3. Discussion

In this study, screening 584 miRNA expressions in rectal NETs revealed that *FBXW7* and mitotic index were the sole candidate genes and clinicopathological features specifically relevant to miRNAs, respectively. *FBXW7* acts as a potent tumor suppressor that may control the expression levels of multiple oncoproteins (i.e., mTOR, notch, cyclin E, c-Myc, and c-Jun) that engage in cellular signaling pathways by inducing them for ubiquitin-mediated proteasomal degradation, thereby protecting against cancer development [27,28]. Several studies have demonstrated that *FBXW7* also significantly influences DNA damage response and repair processes, prime molecular mechanisms for maintaining cellular homeostasis, and genomic integrity [29,30,31,32]. Accordingly, *FBXW7* dysregulation in this process may be a critical factor contributing to tumorigenesis [27,28]. A recent study has reported a high frequency of *P53* (61%), *APC* (53%), *FBXW7* (25%), and *KRAS* (25%) mutations in rectal NETs [15]. Likewise, in our previous study, *FBXW7* (p.R465H) was found to be likely pathogenic in low-grade rectal NETs [18]. In this context, it may be important to focus on the epigenetic regulation of *FBXW7* in rectal NETs, as *FBXW7* is a critical regulator of several key signaling pathways involved in cell growth and proliferation [27,28]. Understanding the epigenetic regulation of *FBXW7* in rectal NETs may provide insights into the mechanisms underlying tumor development and potentially identify novel therapeutic targets for treating these tumors.

Alterations in *FBXW7* expression have been linked to the development and progression of several types of cancer [27,28]. Some *FBXW7* regulators include miRNAs, such as miR-27 in lung cancer, miR-32 in breast cancer, miR-92a in cervical cancer, miR-223 in gastric cancer, miR-182 in lung cancer, and miR-223 in T-cell lymphoma [33,34,35]. In the rectal NETs of the present study, seven miRNAs (hsa-miR-769-5p, hsa-miR-221-3p, hsa-miR-34a-5p, hsa-miR-181c-5p, hsa-miR-1246, hsa-miR-324-5p, and hsa-miR-361-3p) were significantly down-regulated in *FBXW7*-mutant tumors. In gastric cancer, miR-223 acted as an oncogene and negatively modulated *FBXW7* expression, governing proliferation, apoptosis, and invasion [33]. Consistent with this, Notch-mediated activation of miR-223 represses *FBXW7* in T-cell acute lymphoblastic leukemia [34]. In breast cancer cells, miR-32 induces cell proliferation and migration, evading apoptosis by down-regulating *FBXW7* [35]. In cervical cancer cells, an up-regulation of miR-92a causes a down-regulation of FBXW7, thereby stimulating tumor cell proliferation and invasion [36]. miR-182 increases the proliferation of non-small cell lung cancer cells by inhibiting *FBXW7* [37].

Here, we noted that hsa-miR-769-5p was most correlated with *FBXW7* mutation, further linked to ECM-receptor interaction and lysine degradation via the KEGG analysis of target signaling pathways. The ECM is a central element of both normal and tumor tissues, consisting of a complex texture of cross-linked proteins to play a role in resident cells’ hydration, elasticity, and structural organization [38]. It importantly helps in neoplastic proliferation, angiogenesis, progression, and tumor survival [39]. Specific ECM alterations regulating tissue biochemical and biomechanical properties have been described in NETs [40], which may imply that several ECM glycoproteins may contribute to the tumorigenesis of NETs. In a recent study, the up-regulation of six fibrogenic genes (*COL5A2*, *COL1A2*, *COL3A1*, *ITGA5*, *ITGB1*, and *ITGB1*) in pulmonary neuroendocrine carcinoma and the down-regulation of pulmonary neuroendocrine neoplasms were strongly related to no metastasis and overall survival [41]. Only one study has shown that the activation of the lysine degradation pathway injures tumor cell proliferation [42], which is also related to miR-769-5p tumor suppression, as shown in subsequent studies. miR-769-5p/miR-769-3p-mediated inhibition of proliferation causes apoptosis in gastric cancer cells [43]. Another study has shown that miR-769-5p inhibited non-small cell lung cancer proliferation and invasion, indicating that this type of tumor could benefit from miR-769-5p as a diagnostic and prognostic biomarker [44]. hsa-miR-769-5p has also been described to be expressed at low levels in papillary thyroid carcinomas, and the overexpression of hsa-miR-769-5p highly affected the formation of the tumor microenvironment [45], which appears to be consistent with our findings.

Up-regulated miR-3934-5p and down-regulated miR-4286, miR-223-3p, miR-129-5p, and miR-153-3p were characterized in tumors with high mitotic indices, in which miR-3934-5p was the only one statistically significant after adjustment. Further clustering analysis separated two main groups: (1) high mitotic index, grade 2, wild-type *FBXW7*, and wild-type *TP53* and (2) low mitotic index, grade 1, *FBXW7* mutation, and *TP53* mutation. This may indicate that these miRNA contributions were mutually exclusive between the high mitotic index and *FBXW7* mutation in rectal NETs. The KEGG pathway maps also confirmed the different pathway involvement in different miRNAs between *FBXW7*-mutated and highly mitotic-active tumors. hsa-miR-3934-5p may be closely implicated in a high mitotic index, eventually contributing to higher grade 2 tumors in rectal NETs.

In contrast, hsa-miR-769-5p related to *FBXW7* mutation may be involved in grade 1 tumors and is less likely to be involved in tumor aggressiveness. Indeed, a recent study has discovered that WHO grade 3 gastro-pancreatic NETs tend to harbor NETs with lower *FBXW7* mutation frequency than grade 1 and grade 2 NETs [15]. Comparisons of normal pancreatic islets with their matched neoplastic groups disclosed changes in ECM compositions, which also differed depending on histological grade [40,46]. This may explain the finding that the ECM-receptor interaction KEGG pathway (hsa-miR-769-5p) was significantly associated with *FBXW7* mutations and was more likely involved in grade 1 tumors. hsa-miR-769-5p appears to be a possible regulator of *FBXW7*-mutated rectal NETs. These differentially expressed miRNAs could be potential tissue biomarkers for aggressive behavior in rectal NETs.

Our study was limited by a relatively small number of rectal NETs. We did not perform the validation step using quantitative PCR. However, we utilized the advantages of Nanostring technology to identify 24 cancer-related miRNAs out of 584 miRNAs without requiring the amplification of transcripts. We believe that our approach was able to identify low-abundance transcripts that may have been difficult to validate through PCR [47]. Our study focused on identifying candidate miRNAs. In addition, we did not analyze the difference between plasma and tissue miRNAs. miRNAs from tumors may be secreted within membrane vesicles (exosomes) or directly into the blood, indicating that miRNAs play a key role in intercellular communication [48]. Plasma miRNAs communicate the most with the pericardial, adipose, liver, and spleen tissues [49]. As rectal NETs are submucosal tumors, tissue-based analysis of our miRNAs may be more appropriate as they reflect the origin of these tumors. Because some may metastasize, we aim to further investigate the differences in plasma miRNAs between localized rectal NETs and those with metastasis to advance our understanding of these tumors in future studies.

Nevertheless, some conclusions emerged from our results. To our knowledge, this is the first study to identify a correlation between *FBXW7* mutations and miRNAs in rectal NETs. hsa-miR-769-5p was most correlated with *FBXW7* mutation and further linked to ECM-receptor interaction and lysine degradation via a KEGG analysis of target signaling pathways. hsa-miR-3934-5p may be strongly implicated in a high mitotic index, which may eventually contribute to higher grade 2 tumors in rectal NETs, whereas hsa-miR-769-5p related to *FBXW7* mutation may be involved in grade 1 tumors and is likely to play a role in suppressing rectal NET progression.

## 4. Materials and Methods

### 4.1. Patients

The institutional review board of Hallym University Sacred Heart Hospital approved this study (HALLYM 2022-11-006-002). All participants provided written informed consent for the procedure, and the study protocol adhered to the guidelines and regulations of the ethics committee of Hallym University. A search in the electronic database of the pathology department of Hallym University Sacred Heart Hospital disclosed 69 consecutive patients with primary rectal NETs who underwent endoscopic resection in the hospital between 1 June 2005, and 31 July 2015. These patients were included in previous studies [6,50]. We excluded 45 patients whose formalin-fixed paraffin-embedded (FFPE) tissue blocks contained too few tumor cells for the assay or whose FFPE tumor blocks were inadequate for molecular analysis.

### 4.2. Histologic Evaluation

All H&E-stained slides were reviewed by a gastrointestinal and molecular pathologist (M.J.K.) to confirm the diagnosis and evaluate histopathological characteristics. The results of mitotic rates, LVI, and proliferative index established in the previous studies were applied in the present study [6,50].

### 4.3. DNA Extraction and NGS

A tissue microtome (Leica Biosystems, Wetzlar, Germany) was used to obtain two 10-μm sections from every FFPE tissue block on the glass slides. Tumor areas on the glass slides were manually macrodissected from the unstained tissue sections to enrich tumor cell populations > 50%. DNA was extracted and purified using the Ion AmpliSeq Direct FFPE DNA Kit (Thermo Fisher Scientific, Waltham, MA, USA) and QIAamp DSP DNA FFPE Tissue Kit (Qiagen, Hilden, Germany), respectively. The yield of purified genomic DNA was estimated using a Qubit 2.0 Fluorometer and Qubit dsDNA HS Assay Kit (Thermo Fisher Scientific) prior to library preparation for sequencing.

The NGS platform was an Ion Personal Genome Machine Sequencer (Thermo Fisher Scientific). Library preparation for each sample was conducted using the Ion AmpliSeq Library Kit 2.0 (Thermo Fisher Scientific) and Ion AmpliSeq Cancer HotSpot Panel v2 (Thermo Fisher Scientific), following the manufacturer’s protocols, as previously explained [18]. The Ion Torrent platform-specific pipeline software was employed via the variant calling process. Annotation was performed using the Variant Effect Predictor. To compare our data with findings from other cancer genome studies and check for hotspot mutations, we downloaded all cancer genome studies from cBioPortal using the CGDS-R package (R foundation for Statistical Computing, Vienna, Austria). To identify confident putative somatic variants, annotated raw variants were filtered according to the following criteria: (1) non-synonymous single-nucleotide variant or short insertion or deletion in coding regions; (2) coverage ≥ 50× and variant allele frequency (VAF) ≥ 5% or coverage ≥ 1000× and VAF ≥ 3%; and (3) minor allele frequency < 0.1% in the gnomAD and 1000 Genomes Project. The resulting list of variants was manually reviewed and visually confirmed using the Integrated Genomics Viewer (http://www.broadinstitute.org/igv/, accessed on 1 October 2022).

### 4.4. miRNA Extraction and Nanostring nCounter miRNA Expression Assay

The miRNAs were isolated from FFPE tissues using the miRNeasy Mini Kit (Qiagen) according to the manufacturer’s instructions. The concentration was assessed using a NanoDrop spectrophotometer (Thermo Fisher Scientific) [51]. nCounter human v3 miRNA expression assays designed and synthesized by NanoString Technologies (NanoString, Seattle, WA, USA) were performed in this study. The miRNA panel included oligonucleotide tags for 798 human miRNAs (from miRBase v21) and 5 housekeeping mRNAs for reference (*ACTB*, *B2M*, *GAPDH*, *RPL19*, and *RPLP0*). Twenty-five control probes that recognized either synthetic mRNA or miRNA targets were used to monitor the efficiency and specificity of each reaction step. Hybridization was performed with 150 ng of total RNA (16 h at 65 °C) with probe pairs consisting of reporter probes carrying the signal at their 5′ end and capture probes carrying biotin on their 3′ ends. After hybridization, sample cleanup and digital report counts were performed according to the manufacturer’s instructions. Raw data were analyzed using the NanoString nSolver software (version 4.0). Internal positive controls normalized raw data to remove variability in hybridization, and negative controls to subtract the background. In addition, miRNA input levels were normalized using the spike-in normalization method according to the manufacturer’s instructions. The minimum threshold to consider an miRNA for further analysis was 20 counts in at least 10% of the samples. This threshold allowed us to identify 584 miRNAs suitable for analysis.

### 4.5. DNA Intelligent Analysis (DIANA)-miRPath for miRNA Pathway Analysis

Pathway analysis using significantly up-regulated and down-regulated miRNAs was performed using DIANA-miRPath v3.0 [52]. This software allowed us to link miRNAs to experimentally validated target genes from Tarbase, v7.0/microT-CDS v5.0 (https://dianalab.e-ce.uth.gr/, accessed on 1 October 2022) and identifies the putative targeted molecular ‘KEGG’ pathways. As our analysis was hypothesis-free, we used the pathway union option of the miRPath software. The pathway analysis was performed using miRPath v3.0 with an FDR < 0.05, using the Benjamini and Hochberg methods.

### 4.6. Statistical Analyses

The differentially expressed mRNAs and significant pathways were selected based on the *p*-value and FDR analysis [53]. The miRNA expression levels were compared to each clinicopathological parameter using the Mann–Whitney U test, calculated using GraphPad Prism 9 (GraphPad Software, San Diego, CA, USA). The *p*-values were obtained through Fisher’s exact test and χ2 test as enrichment analysis methods. The FDR was estimated using the two-stage process of Benjamini, Krieger, and Yekutieli using FDR < 0.05 and *p* < 0.001 as the significance threshold [26]. Hierarchical clustering was performed using the R package, version 4.13, using Pearson’s correlation.

## 5. Conclusions

Different miRNAs related to high mitotic indices or *FBXW7* mutations may contribute to tumor behaviors in rectal NETs. Specifically, *FBXW7* mutations are likely to play an important role in inhibiting rectal NET progression, whereas mitosis may be the only significant histological risk factor related to miRNA expression. Furthermore, differentially expressed miRNAs may be used as potential tissue biomarkers for possible prognostic indicators in rectal NETs.

## Figures and Tables

**Table 1:** Demographic and clinical features of patients with rectal neuroendocrine tumors.

Characteristic	N = 24
Sex	
Male	17 (70.8%)
Female	7 (29.2%)
Age, mean ± SD (years)	48.5 ± 9.51 (range, 34–73)
≤50	16 (66.7%)
>50	8 (33.3%)
Tumor size, mean ± SD (mm)	7.62 ± 2.81 (range, 5–15)
<10 mm	17 (70.8%)
≥10 mm	7 (29.2%)
WHO grade	
Grade 1	16 (66.7%)
Grade 2	8 (33.3%)
Mitotic count/10 HPFs	
0–1	18 (75.0%)
2–3	6 (25.0%)
Ki-67 labeling index	
<3%	22 (91.6%)
≥3%	2 (8.3%)
Lymphovascular invasion	
Negative	17 (70.8%)
Positive	7 (29.2%)
Mutated detected tumors	
No detected	5 (20.8%)
Detected	19 (79.2%)
*TP53*	
Wild-type	17 (70.8%)
Mutated	7 (29.2%)
*FBXW7*	
Wild-type	19 (79.2%)
Mutated	5 (20.8%)
*CDKN2A*	
Wild-type	20 (83.3%)
Mutated	4 (16.7%)
*PTEN*	
Wild-type	20 (83.3%)
Mutated	4 (16.7%)

SD, standard deviation; HPF, High power field.

**Table 2:** miRNA expression profiles between *FBXW7*-wild-type and -mutated rectal neuroendocrine tumors.

Dysregulated miRNAs	*FBXW7* Wild-Type vs. Mutated		Expression in *FBXW7*- Mutated Tumors
	*p*-Value *	Adjusted *p*-Value **	
hsa-miR-769-5p	0.000047	0.000047	down-regulated
hsa-miR-221-3p	0.000565	0.000565	down-regulated
hsa-miR-34a-5p	0.000565	0.000565	down-regulated
hsa-miR-181c-5p	0.000847	0.000847	down-regulated
hsa-miR-1246	0.000894	0.000894	down-regulated
hsa-miR-324-5p	0.000894	0.000894	down-regulated
hsa-miR-361-3p	0.000894	0.000894	down-regulated

* *p*-values were obtained by using Mann–Whitney *U* test. (significant, *p* < 0.001); ** Adjusted *p*-values using the two-stage method of Benjamini, Krieger, & Yekutieli [26]. (significant, FDR < 0.05).

**Table 3:** Significantly differentially expressed miRNAs between low (0–1) and high (≥2) mitotic index in rectal neuroendocrine tumors.

Dysregulated miRNAs	Mitotic Index (Low vs. High)		Expression in Tumors with High Mitotic Index
	*p*-Value *	Adjusted *p*-Value **	
hsa-miR-3934-5p	0.000040	0.000047	up–regulated
hsa-miR-153-3p	0.003259	0.366977	down-regulated
hsa-miR-4286	0.004207	0.001365	down-regulated
hsa-miR-223-3p	0.006796	0.023951	down-regulated
hsa-miR-129-5p	0.008518	0.235225	down-regulated

HPF, High power field. Mitotic index refers to mitotic counts per 10 HPFs. A low mitotic index is considered 0–1 mitosis per 10 HPFs, whereas a high mitotic index is considered ≥2 mitoses per 10 HPFs. * *p*-values were obtained by using Mann–Whitney *U* test. (significant, *p* < 0.001); ** Adjusted *p*-values using the two-stage method of Benjamini, Krieger, & Yekutieli [26]. (significant, FDR < 0.05).

**Table 4:** KEGG pathway analysis involved in significantly differentially expressed miRNAs between *FBXW7* wild-type and mutated rectal neuroendocrine tumors.

Signaling Pathway	*p*-Value	Target Genes	miRNAs
Fatty acid biosynthesis	1 × 10^−325^	*FASN*, *ACSL4*, *ACSL1*, *ACACA*	hsa-miR-34a-5p hsa-miR-361-3p
ECM-receptor interaction	1 × 10^−325^	*LAMB2*, *LAMB1*, *THBS1*, *COL4A5*, *COL4A2*, *COL6A2*, *COL5A1*, *COL1A1*, *DAG1*, *COL1A2*, *COL4A6*, *TNC*, *SDC4*	hsa-miR-769-5p hsa-miR-361-3p
Fatty acid metabolism	1.67 × 10^−11^	*FASN*, *SCD5*, *ACOX1*, *ACOX3*, *ACAA2*, *PPT1*, *PTPLA*, *CPT2*, *ACADVL*, *PPT2*, *SCD*, *ACSL4*, *ACSL1*, *HSD17B12*, *MECR*, *ACACA*	hsa-miR-34a-5p hsa-miR-361-3p
Lysine degradation	2.49 × 10^−8^	*SETD1B*, *NSD1*, *ASH1L*, *KMT2D*, *DOT1L*, *WHSC1*, *KMT2A*, *KMT2E*, *SETD1A*	hsa-miR-769-5p hsa-miR-361-3p
Viral carcinogenesis	8.07 × 10^−8^	*PIK3CB*, *BAX*, *CDK6*, *TP53*, *KAT2B*, *CCNE1*	hsa-miR-1246
Hippo signaling pathway	3.76 × 10^−5^	*PPP1CA*, *YAP1*, *YWHAG*, *DLG4*, *CCND1*, *PPP2R1A*, *TEAD2*, *PARD6B*	hsa-miR-361-3p
Glioma	4.73 × 10^−5^	*BRAF*, *PDGFRA*, *E2F1*, *CDK4*, *E2F2*, *MAP2K2*, *TGFA*, *PIK3R2*, *RAF1*, *EGFR*, *CDKN2A*, *CDK6*, *ARAF*, *TP53*, *AKT2*, *PLCG1*, *CCND1*, *E2F3*, *PRKCB*, *IGF1*, *PIK3CA*, *CDKN1A*, *MAP2K1*, *PTEN*, *MAPK1*, *PDGFRB*	hsa-miR-34a-5p
Chronic myeloid leukemia	3.82 × 10^−4^	*BRAF*, *E2F1*, *NFKB1*, *CDK4*, *E2F2*, *CRKL*, *MAP2K2*, *CRK*, *RUNX1*, *HDAC1*, *PIK3R2*, *RAF1*, *CDKN2A*, *CDK6*, *TGFB1*, *ARAF*, *TP53*, *AKT2*, *CCND1*, *SMAD4*, *E2F3*, *MYC*, *NFKBIA*, *PTPN11*, *PIK3CA*, *CDKN1A*, *MAP2K1*, *MAPK1*, *TGFBR2*, *BAD*, *TGFB3*	hsa-miR-34a-5p
Adherens junction	4.54 × 10^−4^	*ACTB*, *CSNK2A2*, *MET*, *RAC2*, *CTNND1*, *PVRL2*, *ACTG1*, *TCF7L1*, *IQGAP1*, *PTPRM*, *EGFR*, *VCL*, *SNAI1*, *TJP1*, *SORBS1*, *FYN*, *MLLT4*, *CDH1*, *SMAD4*, *CTNNB1*, *WASF2*, *CSNK2A1*, *FARP2*, *SRC*, *EP300*, *LEF1*, *PARD3*, *FGFR1*, *MAPK1*, *TGFBR2*, *PVRL1*	hsa-miR-34a-5p

ECM, extracellular matrix.

## Data Availability

The data used to support the findings of this study are available from the corresponding author upon request.
